# Emotional Dysregulation and Temperament in Adolescents with Acute Psychiatric Conditions: Gender Differences and the Role of Psychiatric Diagnosis

**DOI:** 10.3390/jcm15031012

**Published:** 2026-01-27

**Authors:** Alessandra Minutolo, Maria Pontillo, Massimo Apicella, Gino Maglio, Giulia D’Amario, Giulia Serra, Giorgia Della Santa, Francesca Boldrini, Milena Labonia, Roberto Averna, Stefano Vicari

**Affiliations:** 1 Child and Adolescent Neuropsychiatry Unit, Bambino Gesù Children’s Hospital, IRCCS (Scientific Institute for Research, Hospitalization and Healthcare), 00165 Rome, Italy; 2 Department of Neuroscience, Catholic University of the Sacred Heart, 00168 Rome, Italy; 3 Department of Life Sciences and Public Health, Catholic University of the Sacred Heart, 00168 Rome, Italy

**Keywords:** emotional dysregulation, adolescents, temperament, gender differences, psychiatric diagnosis, acute psychiatric care

## Abstract

**Background:** Emotional dysregulation (ED) is a transdiagnostic construct implicated in a broad range of psychiatric conditions. However, the influence of gender on ED remains understudied, particularly among adolescents with severe mood and behavioral disorders. Furthermore, few studies have controlled for confounding effects of specific psychiatric diagnoses. **Methods:** We assessed 182 adolescents (80.8% female; mean age 15.7 years) referred to our clinical institution. Participants completed the Cyclothymic–Hypersensitive Temperament Questionnaire (CHTQ), the Reactivity, Intensity, Polarity, and Stability Questionnaire (RIPoSt-Y), and the K-SADS-PL interview. **Results:** Females reported significantly higher levels of CHTQ mood lability (7.53 vs. 5.94, *p* = 0.012), RIPoSt-Y affective instability (62.33 vs. 53.31, *p* = 0.023), and interpersonal sensitivity (30.80 vs. 24.97, *p* < 0.001). They also exhibited higher rates of cyclothymic–hypersensitive temperament (46.6% vs. 14.7%, *p* = 0.001). Regression analysis revealed that gender and specific psychiatric diagnoses exerted significant independent effects on ED dimensions. Mood lability/hypersensitivity was significantly predicted by bipolar disorder (*p* = 0.001), depressive disorder (*p* = 0.002), and female sex (*p* = 0.025). Affective instability was independently predicted by bulimia nervosa (*p* = 0.019), depressive disorder (*p* = 0.004), and female sex (*p* = 0.033). Significant predictors for interpersonal sensitivity included female sex (*p* = 0.002), depressive disorder (*p* = 0.008), bulimia nervosa (*p* = 0.044), and the absence of conduct disorder (*p* = 0.048). **Conclusions:** Female adolescents with severe psychiatric presentations exhibited higher levels of ED, specifically regarding mood lability, affective instability, and interpersonal sensitivity. These associations persisted independently of current mood disorder diagnoses or comorbidities. While findings from this clinical cohort may not be fully generalizable to the general population, they highlight the need for gender-informed clinical interventions for adolescents characterized by severe ED.

## 1. Introduction

Emotional dysregulation (ED) refers to impairments in modulating the intensity and quality of emotions and in generating appropriate emotional responses. This construct encompasses difficulties in managing excitability, mood instability, and emotional overactivity, as well as challenges in returning to an emotional baseline. It is characterized by patterns of emotional experience or expression that interfere with goal-directed activities [1,2,3]. A widely used theoretical framework for ED [4] defines it through dimensions of impaired awareness, understanding, and acceptance of emotions, alongside impaired impulse control and limited access to regulation strategies. Alternatively, from a viewpoint rooted in Akiskal and Hantouche’s clinical perspective, ED represents a core feature of cyclothymic psychopathology and an amplification of cyclothymic affective temperaments [5,6]. Within this temperamental framework, the cyclothymic–hypersensitive temperament—characterized by marked mood instability, emotional hyperreactivity, interpersonal sensitivity, and rapid shifts between opposing affective states—emerges as a key vulnerability factor for persistent ED [7,8].

The Reactivity, Intensity, Polarity, and Stability Questionnaire (RIPoSt-Y), a recently validated instrument for the multidimensional assessment of ED in adolescents, conceptualizes ED within this framework as impairment in emotional reactivity, intensity, polarity, and stability. Integrating these perspectives posits ED as a continuum: ranging from stable traits, evident since early neurodevelopmental stages and rooted in affective temperaments, to severe ED as the primary presenting complaint in many adolescent psychopathological pictures, with significant clinical and prognostic implications [9]. ED is a transdiagnostic factor across a wide range of psychiatric disorders. For instance, it has been associated with increased risks of anxiety, aggressive behavior, and eating disorders in adolescents [10]. Additionally, ED may mediate the relationship between adverse experiences, such as peer victimization, and the development of psychopathological symptoms [11].

ED in adolescence is highly relevant due to its significant impact on mental health and development [2]. Adolescence is a critical developmental period characterized by heightened emotional reactivity and ongoing neurobiological changes in emotion-regulation circuits (e.g., the prefrontal cortex and limbic system) [12]. Neuroimaging studies indicate that adolescence is marked by increasing inverse functional connectivity between the amygdala and the prefrontal cortex, particularly within the dorsomedial and ventrolateral regions [13]. These changes may increase vulnerability to psychopathology, including anxiety, depression, and aggressive behaviors. Conversely, longitudinal studies have shown that adolescents with stronger limbic–prefrontal connectivity, indicative of superior emotion regulation abilities, report fewer internalizing and externalizing symptoms over time [13,14]. Furthermore, treatments addressing ED, such as Dialectical Behavioral Therapy (DBT), are effective for adolescents with self-harm and severe mood disorders [15]. These considerations highlight the importance of reinforcing ED abilities during adolescence to manage current emotional challenges and reduce vulnerability to future mental health disorders.

Gender differences in adolescent psychopathology are well documented, particularly regarding mood disorders and their frequent comorbidities. Research indicates that girls are more likely to experience internalizing symptoms, whereas boys are more prone to externalizing symptoms, including conduct disorders and hyperactivity [16,17,18]. Liu et al. showed that developmental trajectories of internalizing and externalizing symptoms vary by gender, supporting the notion that externalizing problems in boys and internalizing problems in girls represent central symptoms that predict future psychopathology [18]. Furthermore, the incidence of affective disorders shows a gender-by-age interaction: before puberty, boys have higher rates of depressive and anxiety disorders, but this pattern reverses after puberty [19]. In non-clinical samples, girls report significantly higher levels of mental health difficulties and lower subjective well-being than boys between the ages of 11 and 14. This trend persists even after controlling for sociodemographic and resilience factors [13]. In clinical samples, adolescent girls with depressive episodes experience greater overall symptom severity, while males exhibit more externalizing comorbidities [20]. Moreover, even in depression with mixed features, symptoms are more severe in adolescent girls, with more pronounced symptoms of ED [21].

Current literature on ED in adults highlights significant gender differences, indicating that women generally exhibit greater emotional reactivity and more pronounced regulation difficulties than men. A complex interaction between sex hormones and ED has also been suggested; for instance, higher testosterone is associated with decreased dysregulation among female adolescents, though this is dampened by higher relative dehydroepiandrosterone [22]. In a study on adolescents and young adults using the Difficulties in Emotion Regulation Scale (DERS), women with mood disorders exhibited greater alexithymia, ED, and impulsivity [23]. Among adolescents with Borderline personality disorder (BPD), sex significantly affects symptoms, with girls showing higher ED and comorbid internalizing psychopathology [24]. Similarly, studies on adults with BPD indicate higher affective instability in females [25]. In affective disorders, young adult women are also more likely to misinterpret negative faces (e.g., angry faces), a marker of disrupted emotion processing [26].

Psychiatric diagnosis is an important modifier of these patterns. For example, adult male cannabis users reported greater difficulties with overall ED, non-acceptance, goals, impulse control, strategies, and clarity [27]. In cases of Post-traumatic stress disorder (PTSD), girls showed greater symptoms associated with a slower heart rate—a marker of autonomic and emotional dysregulation—while boys did not [28]. Gender differences in ED are evident from childhood through adolescence and are significantly represented in patients with Attention-Deficit/Hyperactivity Disorder (ADHD). Boys with ADHD show a greater reduction in ED, irritability, and anxiety with age compared to girls, whose symptoms tend to remain elevated [29].

Although gender differences in ED have been explored in adults and, more rarely, in adolescents [23], they remain overall underexplored in younger populations. Crucially, these differences have not been sufficiently investigated while controlling for the potentially confounding effects of psychiatric diagnosis and comorbidities. Furthermore, current literature is largely limited to the use of the DERS and the Child Behavior Checklist (CBCL) “deficient emotion self-regulation” index [20,23]. To our knowledge, the RIPoSt-Y has not yet been utilized to explore these differences.

Based on these premises, the present study aims to explore multidimensional gender differences in emotional dysregulation among adolescents, accounting for both current symptoms (measured by the RIPoSt-Y) and highly correlated cyclotymic–hypersensitive temperamental traits (assessed by the Cyclothymic–Hypersensitive Temperament Questionnaire, CHTQ [8]), and to investigate whether gender significantly accounts for the differential expression of emotional dysregulation when controlling for age, psychiatric diagnosis, and comorbidities—factors whose incidence varies by sex and which may account for higher levels of ED.

Given that existing literature has focused on selected clinical populations, such as patients with borderline personality disorder [24,25] or mood disorders [20,23,26,30], we selected a broad clinical sample of consecutive adolescents admitted to our Child and Adolescent Psychiatry services to better account for the confounding role of psychiatric diagnoses.

## 2. Materials and Methods

### 2.1. Participants

The sample comprised adolescents who were admitted to the Child and Adolescent Neuropsychiatry Unit of the Bambino Gesù Children’s Hospital, either as inpatients or through Day Hospital services, between May and November 2024. Access to our high-intensity psychiatric services is provided through a 24/7 open emergency department, as well as via referrals from community care services. Consequently, the study population consists of adolescents presenting with severe mood and behavioral disorders.

We included consecutive adolescents of both sexes, of 12 to 17 years of age, who completed the psychopathological assessment. Exclusion criteria included intellectual developmental disorder (IQ < 70), autism spectrum disorder, Schizophrenia and related disorders according to DSM-5-TR criteria and significant impairment in the comprehension of the Italian language that could interfere with the administration of the assessment tools.

Cross-sectional data were collected. To ensure data reliability, clinical records for each participant were reviewed, and relevant information was summarized using structured data collection forms. These forms were completed independently by two researchers (AM and GDS), with consensus reached in collaboration with a third researcher (MA) to resolve even minor discrepancies.

### 2.2. Assessment

All participants underwent a comprehensive clinical assessment as part of our clinical evaluation protocol. The primary psychiatric diagnosis was established using the Kiddie Schedule for Affective Disorders and Schizophrenia—Present and Lifetime Version (K-SADS-PL), a semi-structured interview administered to the adolescent and at least one parent or legal guardian [31]. This instrument enabled the confirmation of diagnoses according to DSM-5-TR criteria and the identification of psychiatric comorbidities.

IQ was measured using the Wechsler Intelligence Scale for Children—Fourth Edition (WISC-IV) [32] and the Wechsler Adult Intelligence Scale—Fourth Edition (WAIS-IV) [33]. The WISC-IV (ages 6–16 years and 11 months) and the WAIS-IV (ages 16–90 years) are designed to assess cognitive ability. Standard scores (mean = 100; standard deviation = 15) for index scores and full-scale IQ were used in the statistical analyses.

ED was assessed using two complementary self-report instruments.

Cyclothymic–Hypersensitive Temperament Questionnaire (CHTQ). The CHTQ is a 25-item dichotomous (yes/no) self-report questionnaire designed to measure cyclothymic–hypersensitive temperament, a trait profile characterized by heightened emotional reactivity, rapid mood fluctuations, and impulsivity [7]. The instrument includes two subscales: Moodiness/Hypersensitivity, typically associated with internalizing symptoms and interpersonal sensitivity, and Impulsivity/Emotional Dysregulation, which is more closely linked to externalizing behaviors and traits commonly observed in bipolar spectrum disorders and ADHD. Scores equal to or above 15 for females and 17 for males indicate a clinically significant profile suggestive of emotional vulnerability.

Reactivity, Intensity, Polarity, and Stability Questionnaire–Youth Version (RIPoSt-Y). The RIPoSt-Y is a 31-item self-report instrument rated on a 6-point Likert scale, specifically developed to capture multiple dimensions of ED in adolescents. It assesses four domains: Reactivity (speed and intensity of emotional responses), Intensity (magnitude of emotional experiences), Polarity (tendency to oscillate between emotional extremes), and Stability (persistence or variability of emotional states over time). Clinical cut-off values are >55 for affective instability, >22 for emotional reactivity, and >29 for interpersonal sensitivity [34].

Although both instruments assess ED, they are designed for distinct clinical applications. The CHTQ captures temperamental characteristics, which are by definition stable, whereas the RIPoSt-Y reflects presenting symptoms and problems related to deficient emotional regulation.

Furthermore, the Columbia Suicide Severity Rating Scale (C-SSRS), a semi-structured interview, was employed to assess for suicidal ideation and behaviors. Suicidal behaviors were coded according to Posner [35], and non-suicidal self-injury (NSSI) was distinguished in accordance with DSM-5 criteria [36].

Clinical Global Assessment Scale (C-GAS) was used as a clinician-rated measure of global psychosocial functioning over the past two weeks, with scores ranging from 1 (most impaired functioning) to 100 (optimal functioning) [37].

### 2.3. Statistical Analysis

Descriptive statistics were computed for the total sample and stratified by sex. Categorical variables are expressed as counts and percentages, whereas continuous variables are reported as means ± standard deviation (SD). Group comparisons were conducted using Pearson’s χ^2^ test or Fisher’s exact test for categorical variables and independent-samples *t* tests for continuous measures, with effect sizes reported as Cramer’s V for χ^2^ tests and Cohen’s d for *t* tests. To account for the inflation of Type I error rate due to multiple comparisons across the different psychometric dimensions, *p*-values were adjusted using the Benjamini-Hochberg False Discovery Rate (FDR) procedure, with the FDR threshold set at 5% (q < 0.05).

Bivariate associations between psychiatric diagnoses and dimensional measures of ED (CHTQ and RIPoSt-Y) were examined using point-biserial correlations. Subsequently, multiple linear regression analyses were performed to assess the predictive value of sex, age, and diagnostic categories on ED and temperament traits. Results are presented as beta coefficients (β) with 95% confidence intervals, standardized coefficients, and corresponding *p*-values.

All analyses were performed using IBM SPSS Statistics (Version 26.0; IBM Corp., Armonk, NY, USA), with statistical significance set at α = 0.05 (two-tailed).

## 3. Results

### 3.1. Sample Characteristics

The final sample consisted of 182 adolescents (80.8% female; mean age = 15.7 ± 1.4 years). Overall, 67.0% of the sample required admission to high-intensity services via the emergency department. Notably, females exhibited significantly higher psychiatric hospitalization rates than males (47.6% vs. 22.9%, *p* = 0.008).

The cohort was characterized by high levels of ED and a substantial prevalence of self-injurious behaviors. Non-suicidal self-injury (NSSI) was reported by 54.9% of the total sample, with females showing a 2.4-fold higher rate than males (61.9% vs. 25.7%, *p* < 0.001). Suicide attempts were recorded in 22.5% of the sample (23.8% in females vs. 17.1% in males, *p* = 0.396), reflecting a non-significant trend toward higher rates in females.

Affective disorders were the most frequent primary diagnoses: 64.8% of participants met criteria for a Major Depressive Episode, 17.0% for Disruptive Mood Dysregulation Disorder (DMDD), and 44.5% for an Anxiety Disorder. No significant sex differences were found in the prevalence of these conditions. However, significant sex differences emerged for Anorexia Nervosa (Females: 19.7% vs. Males: 5.7%, *p* = 0.047) and externalizing disorders, which were more frequent in males: ADHD (25.7% vs. 10.9%, *p* = 0.022), Oppositional Defiant Disorder (31.4% vs. 12.9%, *p* = 0.008), and Conduct Disorder (11.4% vs. 2.0%, *p* = 0.009). Global functioning was moderately to severely impaired, with no significant differences in C-GAS scores between groups (Males: 54.53 ± 8.53; Females: 54.34 ± 8.87; *p* = 0.911). Detailed demographic and clinical features are presented in Table 1.

### 3.2. Gender Differences in Emotional Dysregulation and Temperament

Female participants exhibited significantly higher ED and hypersensitive temperament traits across multiple dimensions (Table 2). On the CHTQ, females showed significantly higher scores on the Mood Lability/Hypersensitivity subscale (7.53 ± 3.35 vs. 5.94 ± 3.17; *p* = 0.012, ES = 0.49). Furthermore, females were more than three times as likely as males to exceed the clinical cut-off for cyclothymic–hypersensitive temperament (46.6% vs. 14.7%; *p* = 0.001, ES = 0.25).

Regarding the RIPoSt-Y, females scored significantly higher in Affective Instability (62.33 ± 21.38 vs. 53.31 ± 18.66; *p* = 0.023, ES = 0.45) and Interpersonal Sensitivity (30.80 ± 8.20 vs. 24.97 ± 7.26; *p* < 0.001, ES = 0.75). The RIPoSt-Y total score was also significantly higher in females (118.35 ± 33.39 vs. 100.37 ± 28.38; *p* = 0.004, ES = 0.58). After applying the Benjamini-Hochberg correction for multiple comparisons, the following differences remained statistically significant: CHTQ Mood Lability, RIPoSt-Y Interpersonal Sensitivity, RIPoSt-Y Affective Instability, and the proportion of subjects exceeding clinical cut-offs for both CHTQ and RIPoSt-Y Interpersonal Sensitivity.

### 3.3. Associations Between Psychiatric Diagnoses and Emotional Dysregulation

Preliminary biserial correlations showed that Depressive Disorders were positively associated with Mood Lability (r = 0.173, *p* = 0.020), Affective Instability (r = 0.278, *p* < 0.001), and Interpersonal Sensitivity (r = 0.246, *p* = 0.001). Bipolar Disorder was specifically correlated with Mood Lability (r = 0.159, *p* = 0.033). Bulimia Nervosa showed strong associations with Affective Instability (r = 0.220, *p* = 0.003), Emotional Reactivity (r = 0.171, *p* = 0.021), and Interpersonal Sensitivity (r = 0.178, *p* = 0.016). Conversely, negative associations were found between externalizing disorders (ADHD, Conduct Disorder) and dimensions of Interpersonal Sensitivity and Affective Instability (all *p* < 0.05).

### 3.4. Multivariable Predictors of Emotional Dysregulation

Multiple linear regression analyses were conducted to examine the associations between dimensional measures of ED and cyclothymic–hypersensitive temperament with sex, controlling for demographic factors and relevant psychiatric diagnoses. In subsequent parallel analyses, multivariable regression models were fitted with mood lability/hypersensitivity (CHTQ), affective instability, emotional reactivity, and interpersonal sensitivity (RIPoSt-Y) as predicted variables; age, sex, and DSM-5 diagnoses (selected from previous preliminary correlation analyses) were entered as predictors in each model. We controlled for multicollinearity in each model, analyzing the Variance Inflation Factor (VIF), all of which were between 1.008 and 1.387, which is below the conservative threshold of 2.5, indicating that the predictors provided independent information to the models without significant redundancy.

Mood Lability/Hypersensitivity scale (CHTQ). The analysis identified female sex (β = 1.342, 95% CI [0.170–2.515], *p* = 0.025, std. β = 0.158), Depressive disorder (β = 1.811, 95% CI [0.673–2.948], *p* = 0.002, std. β = 0.258), and Bipolar disorder (β = 2.968, 95% CI [1.197–4.738], *p* = 0.001, std. β = 0.264) as significant predictors of mood lability/hypersensitivity. Neither age nor generalized anxiety disorder showed significant associations (Table 3).

Affective Instability scale (RIPoSt-Y). Significant predictors included female sex (β = 8.085, 95% CI [0.664–15.505], *p* = 0.033, std. β = 0.151), Depressive disorder (β = 9.458, 95% CI [3.124–15.791], *p* = 0.004, std. β = 0.214), and Bulimia nervosa (β = 11.221, 95% CI [1.880–20.562], *p* = 0.019, std. β = 0.167). Age approached statistical significance (β = 1.968, 95% CI [−0.114–4.051], *p* = 0.064, std. β = 0.134), whereas Disruptive Mood Dysregulation disorder and ADHD were non-significant predictors (Table 3).

Emotional Reactivity scale (RIPoSt-Y). Bulimia nervosa was the only significant predictor (β = 5.222, 95% CI [0.857–9.587], *p* = 0.019, std. β = 0.174). No other variables, including age, sex, or additional diagnoses, demonstrated significant effects (Table 3).

Interpersonal Sensitivity scale (RIPoSt-Y). Positive predictors included female sex (β = 4.753, 95% CI [1.821–7.685], *p* = 0.002, std. β = 0.225), Depressive disorder (β = 3.327, 95% CI [0.868–5.785], *p* = 0.008, std. β = 0.191), and Bulimia nervosa (β = 3.773, 95% CI [0.099–7.448], *p* = 0.044, std. β = 0.142). Conduct disorder was negatively associated with interpersonal sensitivity (β = −6.118, 95% CI [−12.193–−0.042], *p* = 0.048, std. β = −0.142). Age, Disruptive Mood Dysregulation disorder, and ADHD were non-significant predictors (Table 3).

## 4. Discussion

We investigated the complex interplay between gender, psychiatric diagnosis, and ED in a large clinical sample of referred adolescents. Our findings demonstrate that female adolescents exhibit significantly higher levels of ED and cyclothymic–hypersensitive traits compared to males, despite comparable levels of global functional impairment. Crucially, multivariable regression models revealed that female sex remains a significant independent predictor of mood lability, affective instability, and interpersonal sensitivity, even after controlling for age and the presence of specific psychiatric disorders such as Depressive disorder, Bipolar disorder, and Bulimia nervosa.

The persistence of gender as a predictor suggests that the heightened ED profile observed in girls—characterized by lability, instability, and interpersonal sensitivity—represents a distinct, gender-related phenotypic expression of distress, relatively independent of the primary psychiatric diagnosis. This is consistent with gender differences reported in adult populations [25,26] and replicates, through a robust analytical plan and multidimensional measures, the findings of Di Benedetto et al. [23].

Furthermore, our results expand the understanding of a “female ED profile” by highlighting specific constructs of temperament often overlooked. While previous frameworks (e.g., DERS, CBCL [23,30]) focus primarily on the failure of “top-down” regulatory strategies, the use of RIPoSt-Y and CHTQ allows for an exploration of a “bottom-up,” constitutional vulnerability. This perspective is particularly relevant for adolescents with severe, early-onset mood and behavioral disorders.

This profile can be interpreted through an integrated biopsychosocial lens. Zanella et al. [38] reported that trait emotional intelligence and habitual emotion regulation strategies are supported by shared resting-state neural dynamics indexed by blood-oxygen-level-dependent (BOLD) signal temporal variability. Greater resting-state BOLD temporal variability within the salience and sensorimotor networks was associated with higher trait emotional intelligence and more frequent use of cognitive reappraisal, whereas reduced BOLD variability within the salience network was linked to increased reliance on expressive suppression. When focusing more specifically on sex differences, the higher prevalence of internalizing symptoms in adolescent girls is well-documented in both community and clinical samples [20,38,39,40]. Biological and psychological factors may overlap in explaining this vulnerability. Increased reactivity of the hypothalamic–pituitary–adrenal axis, heightened sensitivity to stressful life events, and a greater prevalence of alexithymic traits [16,39] might explain both the higher levels of ED and the increased prevalence of internalizing disorders in females. Additionally, sex-specific factors such as Premenstrual Dysphoric Disorder may exacerbate affective instability and ED. From a neurobiological standpoint, females may exhibit stronger reactivity to negative emotional stimuli—a “negativity bias” that leads to more intense and prolonged emotional experiences [18], directly linking to the prominent sex differences in interpersonal sensitivity observed in our sample. Furthermore, gender-specific maladaptive strategies, such as rumination, which are more frequently reported by girls [41], likely contribute to the severity of this profile.

As a secondary finding, our study offers insight into how specific ED dimensions align with psychiatric diagnoses. While reinforcing the conceptualization of ED as a transdiagnostic construct, we identified unique clusters. Depressive Disorder was a robust predictor of mood lability and interpersonal hypersensitivity, while Bipolar Disorder showed a more pronounced correlation with cyclothymic temperament. This confirms that while Bipolar Disorder is rooted in a cyclothymic–hypersensitive substrate [5,8], early-onset Major Depression is also characterized by significant dysregulation and instability. This underscores the importance of adopting a dimensional lens; as seen in adult studies, ED exists on a continuum with similar expressions across both unipolar and bipolar disorders [42,43].

Interestingly, Bulimia Nervosa emerged as a powerful predictor across almost all ED dimensions, including emotional reactivity and affective instability. This suggests that eating pathology in adolescence, particularly of the binge-purging type, may function as a maladaptive attempt to regulate overwhelming negative affect [44]. Our findings align with Igra et al. [45], who identified ED as a prominent transdiagnostic trait in eating disorders, and McClure et al. [46], who demonstrated that difficulties in employing adaptive strategies predict the persistence of bulimic behaviors.

Conversely, the lack of association (or negative association) between ADHD/DMDD and the RIPoSt-Y scales suggests a phenomenological distinction. The irritability and impulsivity typical of DMDD/ADHD may differ from the “affective instability” measured by the RIPoSt-Y, which focuses more on the subjective experience of emotional shifts. This also potentially aligns with research suggesting that ED in behavioral disorders is often less rooted in affective distress and more reflective of impaired inhibitory control [47]. However, this interpretation is limited by our sample’s characteristics: the high rate of comorbidity with disruptive/impulse control disorders (from ODD to Conduct Disorder) may make this group less representative of the broader ADHD population, with a presumably higher representation of callous-unemotional traits in this subgroup.

### 4.1. Limitations

As a first limitation of this study, we acknowledge that the sample is not gender-balanced (4:1 female-to-male ratio). While this is a limitation, it reflects real-world patient flow in a real life high-intensity psychiatric care setting, consistently with recent literature on psychiatric emergency settings; after recent COVID-19 pandemic, the number of female adolescents has been significantly increasing in emergency psychiatric settings [48]. As expected in a sample of consecutive patients, the prevalence of specific disorders differs between sexes, with males presenting more externalizing disorders and females more internalizing one, consistently with previous studies in general population and in clinical cohorts [17,49,50]. Our analytical plan including controlling for psychiatric diagnosis with multivariate analyses of the Temperament/ED–sex association provides robust data on the sex differences depicted.

Second, the cross-sectional design precludes causal inferences; longitudinal studies are required to elucidate directionality. Third, our sample consisted exclusively of high-acuity hospitalized adolescents, limiting generalizability to the general population.

### 4.2. Future Directions

The findings of the present study emphasize the importance of adopting a multidimensional approach to ED in clinical settings, integrating categorical diagnoses with transdiagnostic psychopathological dimensions. The persistence of gender differences after adjusting for diagnosis has significant implications for personalized treatment. The higher interpersonal sensitivity and mood lability in girls suggest that interventions focusing on interpersonal effectiveness and distress tolerance, such as Dialectical Behavior Therapy for Adolescents (DBT-A), might be particularly indicated in females across different diagnoses, from depression to eating disorders. For males, the negative correlation with interpersonal sensitivity may indicate a need for treatments addressing dimensions not captured by the instruments used here, such as alexithymia or callous traits. Finally, future research should incorporate multi-informant data, including parent reports measures, and clinician-rated assessments of emotional dysregulation, as well as longitudinal follow-up designs applied across different clinical contexts (acute and outpatient settings), in order to further characterize and deepen the understanding of the identified temperamental and emotional dysregulation profiles.

## 5. Conclusions

In conclusion, female gender emerged as a significant independent predictor of mood lability, affective instability, and interpersonal sensitivity, regardless of primary psychiatric diagnosis. These findings delineate a distinct “female ED profile” that warrants a dimensional, gender-informed approach in clinical practice.

## Figures and Tables

**Table 1:** Demographic and clinical features of adolescents with ED by gender. Means (±standard deviation) are reported for continuous variables; percents are reported for categorical variables. C-SSRS = Columbia Suicide Severity Rating Scale; NSSI = Non-Suicidal self-injury; DMDD = Disruptive Mood Dysregulation Disorder; ADHD = Attention Deficit/hyperactivity Disorder; C-GAS = Children’s Global Assessment Scale.

Variable	Males (n = 35)	Females (n = 147)	Total (n = 182)	*p*-Value
Age (years)	15.75 (±1.31)	15.68 (±1.47)	15.7 (±1.44)	0.793
Referral from an urgent psychiatric evaluation	60.0%	68.7%	67.0%	0.325
Psychiatry inpatient Admission	22.9%	47.6%	42.9%	0.008
Admission Duration (days)	2.43 (±9.81)	3.72 (±7.19)	3.47 (±7.75)	0.378
History of one or more Suicide Attempts	17.1%	23.8%	22.5%	0.396
C-SSRS Screening	1.83 (±2.19)	2.31 (±2.25)	2.21 (±2.24)	0.258
NSSI	25.7%	61.9%	54.9%	<0.001
Depressive Disorder	65.7%	64.6%	64.8%	0.904
DMDD	17.1%	17.0%	17.0%	0.985
Bipolar Disorder	8.6%	10.2%	9.9%	0.771
Adjustment Disorder	8.6%	12.2%	11.5%	0.541
Post Traumatic Stress Disorder	8.6%	6.1%	6.6%	0.600
Anxiety Disorder	37.1%	46.3%	44.5%	0.325
Panic Disorder	2.9%	10.2%	8.8%	0.168
Social Anxiety Disorder	11.4%	20.4%	18.7%	0.221
Generalized Anxiety Disorder	34.3%	44.9%	42.9%	0.254
Separation Anxiety Disorder	8.6%	12.2%	11.5%	0.541
Anorexia	5.7%	19.7%	17.0%	0.047
Bulimia	5.7%	12.2%	11.0%	0.267
ADHD	25.7%	10.9%	13.7%	0.022
Learning Disorder	20.0%	12.2%	13.7%	0.231
Tic Disorder	2.9%	1.4%	1.6%	0.532
Oppositional Defiant Disorder	31.4%	12.9%	16.5%	0.008
Conduct Disorder	11.4%	2.0%	3.8%	0.009
Obsessive Compulsive Disorder	8.6%	6.8%	7.1%	0.715
Substance Use Disorder	14.3%	8.2%	9.3%	0.263
C-GAS	54.53 (±8.53)	54.34 (±8.87)	54.38 (±8.78)	0.911

**Table 2:** Gender differences in emotional dysregulation. Means (±standard deviation) are reported for continuous variables; percents are reported for categorical variables. CHTQ = Cyclothymic–Hypersensitive Temperament Questionnaire; RIPoSt-Y = Reactivity, Intensity, Polarity, and Stability Questionnaire-Youth Version; ES: effect size.

Measure	Males	Females	*p*-Value	ES
CHTQ Mood Lability score	5.94 ± 3.17	7.53 ± 3.35	0.012	0.49
CHTQ Impulsivity score	5.97 ± 2.66	6.23 ± 2.86	0.626	0.09
RIPoSt-Y Interpersonal Sensitivity score	24.97 ± 7.26	30.80 ± 8.20	<0.001	0.75
RIPoSt-Y Affective Instability score	53.31 ± 18.66	62.33 ± 21.38	0.023	0.45
RIPoSt-Y Emotional Reactivity score	22.46 ± 7.63	25.01 ± 9.78	0.099	0.27
CHTQ total > cut-off	14.7%	46.6%	0.001	0.25
RIPoSt-Y Interpersonal Sensitivity score > cut-off	37.1%	63.3%	0.005	0.21
RIPoSt-Y Affective Instability score > cut-off	60.0%	70.7%	0.218	0.09
RIPoSt-Y Emotional Reactivity > cut-off	57.1%	66.0%	0.326	0.07

**Table 3:** Multivariable linear regression models predicting emotional dysregulation dimensions. β = Unstandardized regression coefficient; CI = Confidence Interval. ADHD = Attention Deficit/Hyperactivity Disorder, DMDD = Disruptive Mood Dysregulation Disorder.

Outcome	Predictor	β (95% CI)	*p* (t)	Standardized β
Hypersensitivity/Mood Swings	Bipolar Disorder	2.968 (1.197, 4.738)	0.001 (3.309)	0.264
	Depressive Disorder	1.811 (0.673, 2.948)	0.002 (3.142)	0.258
	Female Sex	1.342 (0.170, 2.515)	0.025 (2.260)	0.158
	Generalized Anxiety	0.932 (−0.010, 1.873)	0.052 (1.954)	0.137
	Age	0.254 (−0.082, 0.591)	0.138 (1.491)	0.109
	Intercept	0.304 (−4.925, 5.532)	0.909 (0.115)	–
Affective Instability	Bulimia	11.221 (1.880, 20.562)	0.019 (2.371)	0.167
	Depressive Disorder	9.458 (3.124, 15.791)	0.004 (2.947)	0.214
	Female Sex	8.085 (0.664, 15.505)	0.033 (2.150)	0.151
	Age	1.968 (−0.114, 4.051)	0.064 (1.865)	0.134
	ADHD	−2.924 (−11.798, 5.950)	0.516 (−0.650)	−0.048
	DMDD	−3.431 (−11.581, 4.720)	0.407 (−0.831)	−0.061
	Intercept	16.793 (−16.725, 50.311)	0.324 (0.989)	–
Emotional Reactivity	Bulimia	5.222 (0.857, 9.587)	0.019 (2.361)	0.174
	Female Sex	2.153 (−1.299, 5.606)	0.220 (1.231)	0.090
	Age	−0.772 (−1.720, 0.175)	0.110 (−1.608)	−0.118
	Intercept	34.326 (19.100, 49.551)	<0.001 (4.449)	–
Interpersonal Sensitivity	Female Sex	4.753 (1.821, 7.685)	0.002 (3.199)	0.225
	Bulimia	3.773 (0.099, 7.448)	0.044 (2.027)	0.142
	Depressive Disorder	3.327 (0.868, 5.785)	0.008 (2.671)	0.191
	Age	0.408 (−0.401, 1.217)	0.321 (0.996)	0.071
	DMDD	−0.766 (−3.956, 2.423)	0.636 (−0.474)	−0.035
	ADHD	−2.172 (−5.640, 1.296)	0.218 (−1.236)	−0.090
	Conduct Disorder	−6.118 (−12.193, −0.042)	0.048 (−1.987)	−0.142
	Intercept	17.531 (4.496, 30.565)	0.009 (2.655)	–

## Data Availability

The data are not publicly available because of ethical concerns.

## Abbreviations

The following abbreviations are used in this manuscript:

ADHD	Attention-Deficit/Hyperactivity Disorder
BOLD	blood-oxygen-level-dependent
BPD	Borderline personality disorder
CBCL	Child Behavior Checklist
C-GAS	Children’s Global Assessment Scale
CHTQ	Cyclothymic–Hypersensitive Temperament Questionnaire
C-SSRS	Columbia Suicide Severity Rating Scale
DBT	Dialectical Behavioral Therapy
DERS	Difficulties in Emotion Regulation Scale
DMDD	Disruptive Mood Dysregulation Disorder
ED	Emotional Dysregulation
IQ	Intelligence Quotient
K-SADS-PL	Kiddie Schedule for Affective Disorders and Schizophrenia Present and Lifetime Version
NSSI	Non-suicidal self-injury
PTSD	Post-traumatic stress disorder
RIPoSt-Y	Reactivity Intensity Polarity and Stability Questionnaire-Youth Version
WAIS-IV	Wechsler Adult Intelligence Scale-Fourth Edition
WISC-IV	Wechsler Intelligence Scale for Children-Fourth Edition
